# Domestic sheep show average *Coxiella burnetii* seropositivity generations after a sheep-associated human Q fever outbreak and lack detectable shedding by placental, vaginal, and fecal routes

**DOI:** 10.1371/journal.pone.0188054

**Published:** 2017-11-15

**Authors:** Ryan D. Oliveira, Michelle R. Mousel, Kristy L. Pabilonia, Margaret A. Highland, J. Bret Taylor, Donald P. Knowles, Stephen N. White

**Affiliations:** 1 Department of Veterinary Microbiology & Pathology, Washington State University, Pullman, Washington, United States of America; 2 USDA-ARS Animal Disease Research, Pullman, Washington, United States of America; 3 Allen School for Global Animal Health, Washington State University, Pullman, Washington, United States of America; 4 Department of Microbiology, Immunology and Pathology, Colorado State University, Fort Collins, Colorado, United States of America; 5 Washington Animal Disease Diagnostic Laboratory, Washington State University, Pullman, Washington, United States of America; 6 USDA-ARS Range Sheep Production Efficiency Research, Dubois, Idaho, United States of America; 7 Center for Reproductive Biology, Washington State University, Pullman, Washington, United States of America; University of Arkansas for Medical Sciences, UNITED STATES

## Abstract

*Coxiella burnetii* is a globally distributed zoonotic bacterial pathogen that causes abortions in ruminant livestock. In humans, an influenza-like illness results with the potential for hospitalization, chronic infection, abortion, and fatal endocarditis. Ruminant livestock, particularly small ruminants, are hypothesized to be the primary transmission source to humans. A recent Netherlands outbreak from 2007–2010 traced to dairy goats resulted in over 4,100 human cases with estimated costs of more than 300 million euros. Smaller human Q fever outbreaks of small ruminant origin have occurred in the United States, and characterizing shedding is important to understand the risk of future outbreaks. In this study, we assessed bacterial shedding and seroprevalence in 100 sheep from an Idaho location associated with a 1984 human Q fever outbreak. We observed 5% seropositivity, which was not significantly different from the national average of 2.7% for the U.S. (P>0.05). Furthermore, *C*. *burnetii* was not detected by quantitative PCR from placentas, vaginal swabs, or fecal samples. Specifically, a three-target quantitative PCR of placenta identified 0.0% shedding (exact 95% confidence interval: 0.0%-2.9%). While presence of seropositive individuals demonstrates some historical *C*. *burnetii* exposure, the placental sample confidence interval suggests 2016 shedding events were rare or absent. The location maintained the flock with little or no depopulation in 1984 and without *C*. *burnetii* vaccination during or since 1984. It is not clear how a zero-shedding rate was achieved in these sheep beyond natural immunity, and more work is required to discover and assess possible factors that may contribute towards achieving zero-shedding status. We provide the first U.S. sheep placental *C*. *burnetii* shedding update in over 60 years and demonstrate potential for *C*. *burnetii* shedding to reach undetectable levels after an outbreak event even in the absence of targeted interventions, such as vaccination.

## Introduction

*Coxiella burnetii* is an important pathogenic bacterium of humans and ruminant livestock that is transmitted by inhalation directly from animals or from a contaminated environment [1–3]. Ruminants are considered the domestic reservoir most responsible for transmission to humans [4]. Environmental persistence of *C*. *burnetii* is accompanied by conversion from the metabolically active large cell variant (LCV) form to the environmentally stable small cell variant (SCV) form, which is believed to contribute to its survival [5] during limiting conditions including high heat or high osmolarity solutions [6]. Since *C*. *burnetii* can persist in the environment for extended periods [7, 8] and since wind can play an important role in transmission [9–11], tracing the source of transmission can be complicated. Nonetheless, sheep have been particularly implicated in *C*. *burnetii* outbreaks within the United States [12, 13]. The bacteria localize within placental trophoblasts and are shed with the placenta in high amounts during parturition [14–16]. In particular, placentas from sheep, goats, and cattle can contain as many as a billion organisms per gram [17–19], and the organism has been detected in the environment of livestock birthing areas [16, 20–22]. In small ruminants, *C*. *burnetii* infection can result in abortions or moribund lambs or kids [14], and costs to the small ruminant industry alone are estimated in excess of a million dollars per year [23].

Disease in humans from *C*. *burnetii*, termed Q fever, occurs in approximately half of infected individuals [24] and is characterized by an acute fever that less commonly includes hepatitis and pneumonia [25, 26]. In a subset of human patients, chronic infection can progress to fatal endocarditis or a fatigue syndrome [27], and seropositivity in women confers higher risk of adverse pregnancy outcomes [28]. Treatment includes protracted antibiotic therapy (between two weeks and a year) with doxycycline and, in chronic cases, hydroxychloroquine [29]. The estimated human infective dose is one bacterium [30]. Human infections are believed to occur by inhalation of birthing- or dust-associated *C burnetii* particles, and the organism is recoverable from the air of cattle [31], sheep [31], and goat [32] pens. Furthermore, several past human outbreaks have been associated solely with common exposure to dust or bedding material from ruminant pens [2, 33–35]. Under wind-driven conditions, transmission can occur over distances of up to several kilometers [9–11].

In a recent outbreak in the Netherlands, a region with small ruminant dairies experienced *C*. *burnetii*-associated abortion storms in 2005–2007 which were followed in 2007–2011 by more than 4,100 human Q fever cases [36, 37]. Many of the human Q fever cases had no direct animal contact but were located within 2 kilometers downwind of the geographic area containing sheep and goat dairy farms, and cases were localized to conditions favorable to airborne dispersal [38]. The Netherlands Q fever outbreak was preceded by endemic *C*. *burnetii* in small ruminants for decades, and there have historically been long periods between recorded Q fever outbreaks [39]. Total costs of controlling the 2007–2011 Netherlands *C*. *burnetii* outbreak were estimated at €307 million, with substantial costs to both small ruminant industry (€85 million) and to broader society (€222 million) [36]. Since *C*. *burnetii* is endemic in most of the world (except New Zealand), there is concern about large outbreaks that could occur potentially at any site throughout its distribution [40]. In the U.S., many of the occupationally related Q fever outbreaks have occurred among biomedical research facilities with exposure to infected pregnant ewes [41]. However, the issue of sheep *C*. *burnetii* shedding outside of current or recent outbreaks has been minimally addressed in field studies.

Seroprevalence of *C*. *burnetii* in sheep has been estimated at 2.7% as of 2011 in the U.S. by the National Animal Health Monitoring Service (NAHMS) (NAHMS, personal communication]. Since recent PCR assessments of organism presence, a direct measure of shedding, have been limited to pooled bovine bulk tank milk or environmental [7] samples, there has been a need for detailed estimates of ruminant shedding. This need is particularly pronounced in sheep from which the placental shedding route has not been investigated in the U.S. since 1951 [17]. Further, little information is available on the long-term ability of premises with presumed endemic *C*. *burnetii* to become free of shedding. While individual goats and sheep have been confirmed to shed *C*. *burnetii* in vaginal fluid for two parturitions after initial infection in an experimental setting [42, 43], persistence of shedding more than 2–5 years after abortion is not well-documented in an extensive rangeland production environment. To date, no study has documented that a flock can demonstrate undetectable levels of placental shedding following a confirmed outbreak of Q fever.

In 1984, 18 cases of human Q fever occurred in Idaho, including four cases requiring hospitalization [44]. Epidemiology involving all cases with available information tied them to contact with a large outdoor Idaho sheep research facility [44]. In the present study, we aimed to provide an updated *C*. *burnetii* seroprevalence and incidence of shedding in placental, vaginal swab, and fecal samples from 100 sheep at the same Idaho location. Placentas were chosen specifically given a dearth of recent studies examining placental shedding in the U.S. and recent data suggesting they are the most sensitive measure of *C*. *burnetii* shedding [45]. Given the premises’ location in the U.S., we made straightforward hypotheses that: 1) seroprevalence in this flock will be equal to the U.S. national average of 2.7%, 2) seroprevalence will be greater than shedding prevalence, and 3) placental shedding will be greater than vaginal or fecal shedding.

## Materials and methods

### Animals and samples

Samples were collected from 100 ewes from 3 breeds (33 Suffolk, 33 Rambouillet, and 34 Polypay) during the spring 2016 lambing season at the same sheep research station in Dubois, ID as previously described [44]. The average age was 2.4 years and four ewes with dead lambs were selectively included to increase the probability of identifying C. burnetii shedding. Whole placentas were collected and triple-bagged prior to freezing. For vaginal swabs, sterile polyester swabs with plastic shafts were inserted approximately 4–6 cm into the vagina, sealed, and stored dry. Prior to testing, 500ul of PCR-grade water was added to the samples. Feces was digitally removed from the rectum and stored dry. All placental samples, vaginal swabs, fecal samples, and blood samples were stored at -20 degrees Celsius within 2–4 hours following parturition. Blood samples were collected by jugular venipuncture using 10 mL vacutainer serum tubes (BD Medical). Sera were removed following centrifugation and stored at -20 degrees Celsius. All animal care and use procedures were reviewed and approved by the Washington State University Institutional Animal Care and Use Committee (Protocol 4710) and/or by the Range Sheep Production Efficiency Research Animal Care and Use Committee (Protocol 16–05). All efforts were made to minimize any discomfort during sample collection.

### Calculation of average pedigree generations

Pedigree and lambing records of sheep at the Idaho location since 1984, the year of the Q fever outbreak, were examined to determine the number of generations that had elapsed between ewes that lambed in 1984 and the 100 selected ewes of the present study. A tail-female pedigree was obtained for each ewe to calculate the average number of generations elapsed in the tail-female line since those that lambed in the 1984 outbreak. In cases where multiple female ancestors lambed in 1984, a simple average of the number of generations back was calculated for each present ewe prior to calculation of the overall population average.

### Diagnostic measures

Placental tissue, vaginal swab, and fecal samples were tested for *Coxiella burnetii* by real-time quantitative PCR (qPCR) at the Veterinary Diagnostic Laboratory of Colorado State University. DNA was purified from placental tissues and vaginal swabs using the QIAamp DNA Blood Mini Kit (Qiagen). Samples were processed according to manufacturer’s instructions, with one exception–an increased 56°C incubation from 10 minutes to 15 minutes. Placental swab samples were mechanically homogenized prior to the extraction process using a Mini-Beadbeater (Biospec Products). DNA was purified from fecal samples using the ZR Fecal DNA MiniPrep kit (Zymo Research), according to manufacturer’s instructions.

DNA from the placental tissues was assayed by qPCR using the Laboratory Response Network (CDC) protocol for *C*. *burnetii*. This qPCR contains three primer/probe sets targeting three different regions of the genome, including both multi-copy and single-copy genes, which provide data to estimate *C*. *burnetii* genome copy number. DNA from the vaginal swab and fecal samples was assayed by a qPCR with a single primer/probe set targeting the multi-copy IS1111 transposon, using TaqMan Universal PCR master mix (Applied Biosystems) [46, 47]. Both qPCRs were performed using the 7500 Fast real-time PCR platform (Applied Biosystems).

Serum samples were analyzed using a CHEKIT Q Fever Antibody ELISA Test Kit (IDEXX) at the Washington Animal Disease Diagnostic Laboratory using the manufacturer-recommended protocol including Phase I and Phase II purified antigens. The sample/positive control (S/P) ratio of optical densities was recorded with a spectrophotometer, and according to manufacturer’s guidelines, each sample was considered ELISA positive if the ratio was 40% or more, indeterminate if between 30% and 40%, and negative if below 30%.

The Clopper-Pearson exact method was used for calculation of 95% confidence intervals because it has been shown to be consistently conservative, even for proportions near zero or 100% [48]. Comparison of the serological positive fraction to national averages where underlying positive count data were unavailable was performed by exact binomial proportion test. Comparisons between *C*. *burnetii* positive fractions from different diagnostic tests or different populations with underlying positive count data were performed with Fisher’s exact test. Results with P<0.05 were considered significant.

## Results

No abortion storms or recent human Q fever diagnoses were observed at this location in 2016 or in records available from the preceding ten years. Of the 100 ewes selected for testing, serum was available from all ewes, placentas from 97 ewes, vaginal swabs from 99 ewes, and fecal samples from 96 ewes. Twin placentas were available from 25 ewes, and triplet placentas were available from one ewe. A minimum of two sample types were available for shedding analysis by PCR from each of the 100 selected ewes. Of the 100 selected ewes, at least one member of the tail-female line from 98 ewes had lambed during 1984, the year of the outbreak. Between 6–15 generations, with an average of 9.21 generations, had elapsed between each ewe and ancestors in the tail-female line that had lambed during 1984.

**Fig 1:**
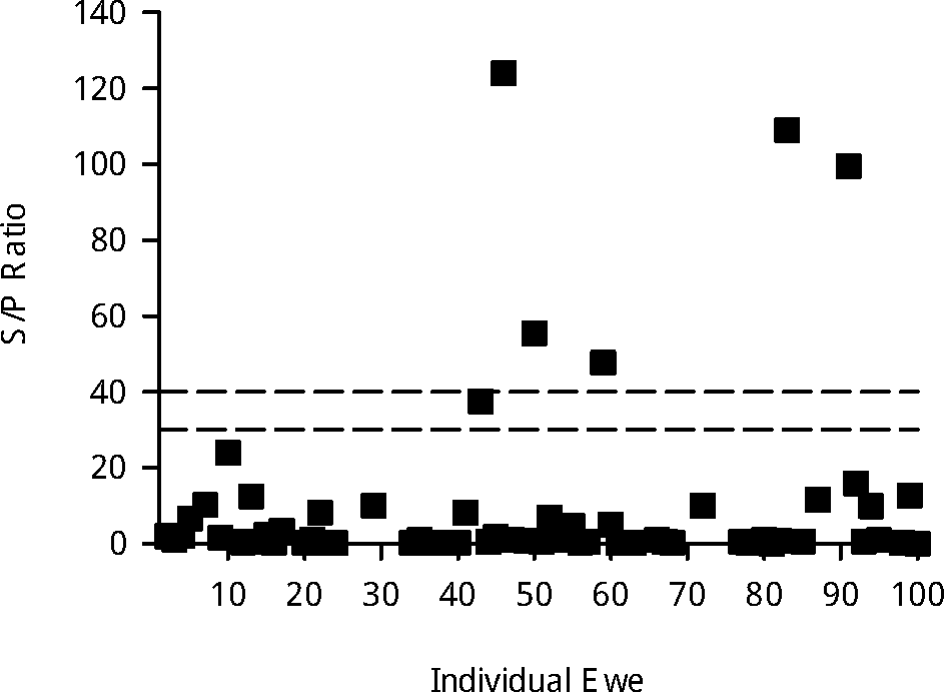
Sample/positive ratios of sera from 100 ewes. Ratios of the sample optical density to positive optical density (S/P ratio) as obtained by *C*. *burnetii* antibody ELISA (CHEKIT Q Fever Antibody ELISA Test Kit, IDEXX). Dashed lines indicate the lower and upper limits of the indeterminate range, as recommended by the manufacturer. Positive samples are represented above the top line, indeterminate samples in between the lines, and negative samples below the bottom line.

Of the serum samples, 5 of 100 had S/P ratios considered positive for *C*. *burnetii* exposure (Fig 1). One serum sample had an indeterminate (suspect) result and was not counted as a positive sample. These data give a 95% confidence interval of 1.6–11.3% (Table 1) for the true population seroprevalence. Comparison of observed seroprevalence to the national average of 2.7% was not significantly different (P>0.05). Using the conservative assumption that the indeterminate ELISA result was actually a seropositive sample, the seroprevalence would be 6/100 (95% CI: 2.2–12.6%), which was also not significantly different than the national average of 2.7% (P>0.05). *C*. *burnetii* was not identified in any placenta, vaginal swab, or fecal samples by qPCR (Table 1). Comparisons of placental shedding versus seropositive fractions were significantly different (P = 0.017), even if the indeterminate serum sample were considered positive (P = 0.0073). Each comparison of shedding fraction between sample types (placental, vaginal, fecal) were nonsignificant (P>0.05).

**Table 1:** Analyses of coordinated samples from 100 ewes by *C*. *burnetii* ELISA and qPCR.

Testing method	# positive/# tested	95% confidence intervals for shedding percentage
Placenta qPCR	0/124	0–2.9%
Vaginal swab qPCR	0/99	0–3.7%
Fecal qPCR	0/96	0–3.8%
Serum ELISA	5/100	1.6–11.3%

## Discussion

In this study, we assessed seroprevalence and shedding of *Coxiella burnetii* in a sheep flock that was epidemiologically linked to human Q fever cases in 1984 [44]. In the time since this occurrence, thousands of ewes were consistently maintained on the premises with new ewes recruited from lambing at this location, though obviously no ewes from the initial outbreak remained on the premises at the time of this study. In the last ten years, animals from outside the location were restricted to introduction of purchased rams and infrequent introduction of purchased ewes. Given the large flock size, experimental evidence that *C*. *burnetii* can be shed for multiple parturitions, extremely high ratio of placental shedding to infectious dose, and environmental persistence of *C*. *burnetii* [5], ongoing intra-flock transmission over time and current shedding of *C*. *burnetii* remained a distinct possibility 32 years later. However, no detectable *C*. *burnetii* DNA was found by qPCR of placentas, feces, or vaginal swabs, and only 5 to 6 of 100 animals showed evidence of prior exposure (seroconversion).

Seroprevalence to *C*. *burnetii* among sheep in large operations (>500 sheep) from sheep production facilities over the United States was estimated at 2.8% by the National Animal Health Monitoring System (NAHMS) in 2011 (USDA APHIS, personal communication). The 5% seroprevalence observed in the current study was not significantly different from this estimated national average (P>0.05). In addition, more localized individual studies of *C*. *burnetii* exposure in domestic sheep within the U.S. between 1951 and 1985 have reported 5.7% [49], 21.2% [50], and 24% [51] seroprevalence, which some have used to generate an average of 16.5% [12]. The lattermost study included a California research facility tied to a human epidemic with a specific seroprevalence of 77% [51]. The observed seroprevalence of 5% (or 6%, if including the indeterminate sample) was significantly lower than each of those values (P<0.05) except the 5.7% figure (P>0.05). The lack of significant difference between seroprevalence in the observed flock (5%) and the recent national average of 2.8% is notable, particularly in light of the high seroprevalence found in a California location associated with human Q fever. The source of exposure is not known for the few sheep that were seropositive, with possibilities including periodically introduced animals from Idaho and other states across the U.S. or vestiges of bacteria on the premises.

No *C*. *burnetii* DNA was detected in all placental, vaginal, or fecal samples in spite of the previous outbreak of Q fever epidemiologically attributed to this facility [44]. While fecal samples may have some degree of PCR inhibition [52], the lack of findings in all three types of samples is striking given, particularly in the placentas, which a previous study found to be the most sensitive route of detection [45]. The prevalence of serum antibody levels to *C*. *burnetii* was significantly greater than prevalence of placental shedding (P = 0.017). This finding is in agreement with previous studies demonstrating seroprevalence as an overestimate of shedding, since seropositive ruminants may not shed detectable *C*. *burnetii* [45, 53, 54]. This result was unable to answer our hypothesis about placental shedding being greater than vaginal or fecal routes since there was no detectable shedding by any measure. No evidence of *C*. *burnetii* infection in either animals or humans was reported on the premises in records available from the ten years preceding this study. While shedding prevalence of *C*. *burnetii* has been previously investigated, no studies of placental shedding in sheep have been conducted within the U.S. in half a century [17]. Additionally, this study is the first known comparison of placental, vaginal, or fecal shedding measures with matched serologic samples of sheep within the U.S.

Sheep and goats experimentally infected with *C*. *burnetii* have been demonstrated to shed for two parturitions following initial abortion, but factors behind progression to lack of *C*. *burnetii* shedding remain mostly unknown. Elimination of large-scale shedding from primarily dairy goat farms affected by the Netherlands outbreak is attributed to a combination of strict hygiene protocols, surveillance, and vaccination [55]. Two studies in sheep flocks following abortions have demonstrated short-term elimination of shedding in adults [56] or complete seronegativity of lambs [57], following extensive intervention, including vaccination, over two years. However, no *C*. *burnetii* vaccine is licensed in the U.S. Short-term elimination of shedding has been documented in a German flock after six years [45] with unknown factors influencing elimination. A previous review has noted that, years after an initial abortion with *C*. *burnetii*, the affected sheep are no longer infective to other sheep, implying a possible loss of shedding, again with unknown contributing factors [58]. In the present study, one contributing factor may have been time passed since the outbreak, which clearly mattered in studies measuring time under a decade [45, 56, 57]. In addition to years, another useful measure of time is generations, for which purpose the pedigree analysis was conducted. A tail-female line was used given the evidence that the placental route is a source of high-level aerosol shedding [1, 20], with spread to the fetus hypothesized to occur through the placenta [14]. Studied sheep were on average 9.21 generations removed from tail-female ancestors that lambed during the epidemic.

Specific changes made at the outbreak location may also have contributed to the lack of demonstrable shedding in the current study. While no antibiotics were used after the outbreak, the demolition of the majority of the original lambing facilities and construction of a new facility, along with subsequent changes made in facility function over the ensuing decades, may have had an effect on the amount of residual *C*. *burnetii* in the lambing area. The facility currently houses approximately 1,428 mature breeding ewes, decreased from 5,270 in 1984, with more than 20,000 acres of land plus. The land use consists of a seasonal schedule of pasture grazing, housing in feedlots for breeding, and winter lots for holding and lambing that have remained invariant or changed only mildly since the outbreak. Changes in husbandry may have reduced infection from the environment over time, and future work on the impact of management conditions on *C*. *burnetii* shedding may clarify which interventions are most helpful. In terms of fetal infection, elimination over subsequent generations may be possible. DNA from *C*. *burnetii* can be detected in the fetuses from infected small ruminant dams [14], but this is not reliably associated with histologically detectable lesions in aborted goat fetuses [59], and it is unknown how or if surviving offspring maintain infection as adults. Future work will be necessary to elucidate the importance of factors that comprise the transition from an abortion outbreak to no detectable shedding. Still, the progression to lack of shedding within the flock examined in this study is notable and is the first known to the authors to be documented in the U.S.

While there were a small number of seropositive sheep identified in this study (5 of 100), implying exposure to *C*. *burnetii* prior to sample collection, we demonstrated lack of *C*. *burnetii* shedding decades after a small Q fever outbreak on the premises. Further characterization of long-term shedding in healthy flocks within the U.S. will better contextualize the lack of shedding observed in this study. Owing to the potential for a zoonotic event, studies are warranted assessing the ability of premises with current *C*. *burnetii* shedding to progress to a zero-shedding flock and the mechanisms by which this can occur.

## Supporting information

S1 Table Results from qPCR and serology by ewe study ID. A list of each ewe’s study ID with the results of the serum S/P ratio and qPCR results for vaginal swabs, feces, and placentas.
(XLSX)
